# High elasticity and strength of ultra-thin metallic transition metal dichalcogenides†

**DOI:** 10.1039/d1na00225b

**Published:** 2021-05-24

**Authors:** Ali Sheraz, Naveed Mehmood, Mert Miraç Çiçek, İbrahim Ergün, Hamid Reza Rasouli, Engin Durgun, Talip Serkan Kasırga

**Affiliations:** Department of Physics, Bilkent University Ankara 06800 Turkey kasirga@unam.bilkent.edu.tr; Institute of Materials Science and Nanotechnology – UNAM, Bilkent University Ankara 06800 Turkey; Department of Engineering Physics, Faculty of Engineering, Ankara University Ankara 06100 Turkey

## Abstract

Mechanical properties of transition metal dichalcogenides (TMDCs) are relevant to their prospective applications in flexible electronics. So far, the focus has been on the semiconducting TMDCs, mostly MoX_2_ and WX_2_ (X = S, Se) due to their potential in optoelectronics. A comprehensive understanding of the elastic properties of metallic TMDCs is needed to complement the semiconducting TMDCs in flexible optoelectronics. Thus, mechanical testing of metallic TMDCs is pertinent to the realization of the applications. Here, we report on the atomic force microscopy-based nano-indentation measurements on ultra-thin 2H-TaS_2_ crystals to elucidate the stretching and breaking of the metallic TMDCs. We explored the elastic properties of 2H-TaS_2_ at different thicknesses ranging from 3.5 nm to 12.6 nm and find that the Young's modulus is independent of the thickness at a value of 85.9 ± 10.6 GPa, which is lower than the semiconducting TMDCs reported so far. We determined the breaking strength as 5.07 ± 0.10 GPa which is 6% of the Young's modulus. This value is comparable to that of other TMDCs. We used *ab initio* calculations to provide an insight into the high elasticity measured in 2H-TaS_2_. We also performed measurements on a small number of 1T-TaTe_2_, 3R-NbS_2_ and 1T-NbTe_2_ samples and extended our *ab initio* calculations to these materials to gain a deeper understanding on the elastic and breaking properties of metallic TMDCs. This work illustrates that the studied metallic TMDCs are suitable candidates to be used as additives in composites as functional and structural elements and for flexible conductive electronic devices.

## Abstract 2

Breaking and elasticity of metallic two-dimensional materials has been overlooked so far. Here, we study four atomically thin metallic transition metal dichalcogenides using atomic force microscopy based nanoindendation and *ab initio* methods.

## Graphical abstract

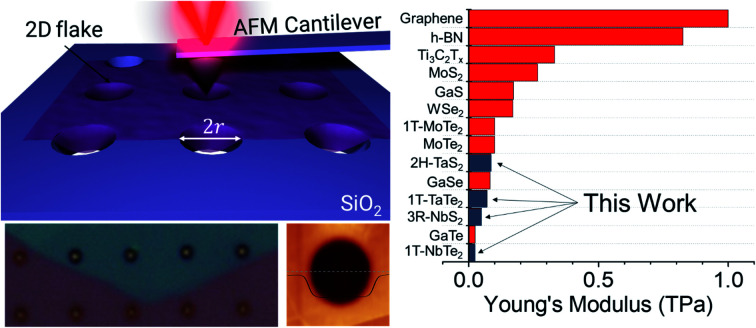

Two dimensional (2D) layered materials show exceptional mechanical strength along the basal plane direction. Ultra-thin crystals of graphene,^1^ h-BN,^2^ MoS_2_ (ref. 3–5) and Ti_3_C_2_T_*x*_ (ref. 6) have record high biaxial Young's modulus with the breaking strength at the intrinsic limit.^7^ Such a level of elasticity and strength in these materials is crucial for the applications in flexible electronics. One particularly appealing family among the 2D layered materials for the prospective applications has been transition metal dichalcogenides (TMDCs). TMDCs are composed of layers formed by covalently bonded transition metals and chalcogen atoms (sulfur, selenium, tellurium) and these layers are stacked *via* van der Waals interactions. At the monolayer limit, MoX_2_ and WX_2_ (X = S, Se) display intriguing optoelectronic properties and attracted a great deal of attention.^8^ Among the TMDCs, so far the mechanical properties of ultra-thin MoS_2_,^3–5^ WS_2_,^9^ WSe_2_,^10^ MoSe_2_,^11^ and MoTe_2_ (ref. 12) has been investigated. Although there are reports on the elastic properties of the bulk samples of other TMDCs, metallic TMDCs are overlooked, and the reported elastic properties are not intrinsic to the materials but dominated by the defects at the grain boundaries and pre-existing cracks.

Here, we elucidated the elastic properties of 2H-TaS_2_, a prototypical metallic TMDC, using atomic force microscopy (AFM) based nanoindentation and supported our findings with *ab initio* calculations. We performed comprehensive measurements on 5 to 19 monolayers of 2H-TaS_2_. We also performed nanoindentation on a small number of 1T-TaTe_2_, 1T-NbTe_2_ and 3R-NbS_2_ crystals and performed *ab initio* calculations. All the materials except 3R-NbS_2_ are exfoliated from the bulk crystals in the ambient using a sticky tape over a PDMS stamp and transferred over circular holes drilled using focused ion beam (FIB).^13^ We used a chemical vapor deposition (CVD) method to grow thin layers of 3R-NbS_2_ and^14^ transferred them over the holes using a wet transfer method (details are provided in the ESI, Fig. S1–S4†). The 3R-polytype of NbS_2_ has the same crystal structure as the monolayer but different stacking in the multilayer. The details of the exfoliation and the CVD synthesis are given in the ESI.† The polytype of each material is confirmed using Raman spectroscopy,^15,16^ shown in Fig. S5.† Optical microscope micrographs of exemplary crystals over the indentation substrates are shown in Fig. S6.†

First, we would like to focus on our measurements on 2H-TaS_2_. 2H-TaS_2_ shows a thickness dependent enhancement in the superconducting transition temperature (*T*_C_ ≈ 0.5 K) and becomes a 2D superconductor below ∼10 nm.^17^ It is well known that both the charge density wave transition and the superconductivity in bulk 2H-TaS_2_ is effected by hydrostatic pressure on the crystal.^18^ Moreover, with a 5.6 eV work function, it is a suitable candidate for rectifying contacts to semiconducting TMDCs.^19^ Thus, it is imperative to understand the elastic properties of metallic 2H-TaS_2_ for possible device applications in low temperature and flexible electronics. 2H-TaS_2_ is relatively difficult to exfoliate and the thinnest crystal we could measure was 5 monolayers thick (with ∼0.7 nm monolayer thickness). 26 samples of 2H-TaS_2_ are measured in total with thicknesses ranging from 3.5 nm to 12.6 nm. A single hole is measured in each sample to reduce the exposure to the ambient. The crystal thicknesses are determined with respect to the supporting substrate surface *via* AFM scan.

A schematic of the measurement setup is given in Fig. 1a. Diameter of the holes etched on the SiO_2_ surface for suspending the ultra-thin crystals is measured as *r* = 1 μm using scanning electron microscope (SEM), which is consistent with the radius determined from the AFM measurements. Optical microscope image of a TaS_2_ crystal transferred over the substrate is shown in Fig. 1b. The AFM cantilevers used for the indentation studies have the spring constant *k* = 40 N m^−1^ and the tip radius *r*_tip_ = 10 nm with diamond like coating, specified by the manufacturer. We preferred a stiffer cantilever to be able to indent the crystals as the thinnest crystal we could find was 5 monolayers. The details regarding the calibration of the AFM cantilevers' spring constant and deflection sensitivity are given in the ESI and Fig. S7.†^20^ To preserve the sample properties, we limited the device fabrication duration to a few minutes, and we finished the indentation measurements within an hour after the crystal transfer. However, we did not observe any statistically significant difference between the Young's moduli obtained from the shorter and the longer duration measurements for 2H-TaS_2_. Dummy samples are scanned for at least half an hour before the indentation measurements to minimize the piezo drift in the tapping mode. Then, the actual sample is scanned, and the tip is positioned at the center of the chosen hole for the indentation studies. We applied gradually increasing forces on the crystals till the brittle fracture.

**Fig. 1:**
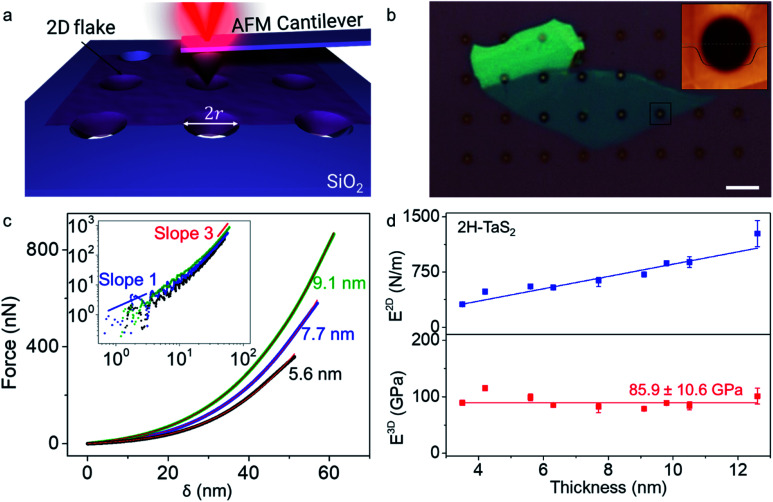
(a) Schematic of the indentation setup is depicted in the illustration. 2D flake is laid over a hole of radius *r* etched over the SiO_2_ surface. (b) Optical microscope micrograph of a 2H-TaS_2_ flake transferred over the holes on oxidized Si chip is shown. AFM height map taken from the black square is given in the inset. Thickness of the crystal at the scan area is 6 nm. Height trace shows that the crystal adheres to the sides of the hole. Scale bar is 5 μm. (c) Force–deflection curves (*F*–*δ*) for 2H-TaS_2_ crystals at different thicknesses, 5.6 nm, 7.7 nm and 9.1 nm. The colored curves are the experimental data and the red solid lines are fit to eqn (1). Inset shows the log–log plot with linear response of the crystal in the first few nanometers of indentation and approaches to the cubic response at the higher loads. (d) 2D elastic moduli (*E*^2D^) and Young's moduli (*E*^3D^) for TaS_2_ at different thickness are given in the plot. Each data point is determined from multiple measurements from a total of 26 different crystals. The average Young's modulus is 85.9 ± 10.6 GPa as denoted with the red line through the graph. *E*^2D^ increases linearly with the thickness similar to other TMDCs.

The force *vs.* displacement (*F*–*δ*) curves obtained from the indentation measurements are fitted using the formula for an elastic membrane under nonlinear deformation:^1,4^1
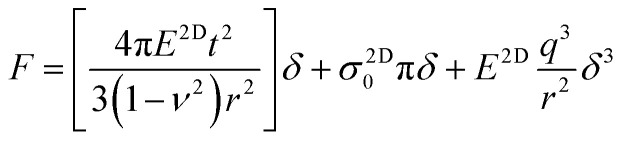
where *E*^2D^ is the 2D elastic modulus, *σ*^2D^_0_ is the prestress in the membrane, *r* is the radius of the hole, *q* is a dimensionless constant related to the Poisson's ratio *ν* as *q* = 1/(1.049 − 0.15*v* − 0.16*v*^2^) and *t* is the thickness of the crystal. Here, the first term represents the stiffness of a plate with a certain bending rigidity and becomes significant for thicker crystals. In our case, as the thickness of the crystals are considerably smaller than the radius of the hole, it is ignorable. The second term is the pretension of a stretched membrane under force and the last term is the nonlinear membrane behavior. The last term is dominant at large loads. Before fitting the measurement data with eqn (1), we determined the point where both the force and displacement are zero by intersecting the line extrapolated from the zero-force line in the *F*–*δ* curve with the curved section.^6^ We checked some of our results with a revised formula that eliminates the need for determination of the zero force and displacement point (ZDP) by fitting the zero force and the membrane displacement parameters.^21^ We obtained identical results with both methods within the error margins. Very large coefficient of determination, *R*^2^ > 0.998 are achieved in fitting all the measurements with eqn (1). Fig. 1c shows the *F*–*δ* curve for crystals with three different thicknesses and the fit of the experimental data with eqn (1). Repeated loading–unloading curves follow the same *F*–*δ* curves show that there is no slippage of the crystals over the holes, even for the ones near the edge.

We measured that 2H-TaS_2_ has a thickness independent Young's modulus value of 85.9 ± 10.6 GPa with the Poisson's ratio of *ν*_TaS_2__ = 0.27 determined by *ab initio* calculations. Young's modulus values for different thicknesses are given in Fig. 1d. Measurements taken from different samples of the same thickness are averaged (individual data points are provided in ESI, Fig. S8†). To best of our knowledge, the room temperature Young's modulus for 2H-TaS_2_ has not been reported in the literature experimentally. Based on the velocity of ultrasonic waves measured in 2H-TaS_2_ below 65 K,^22^ we estimated the Young's modulus of 2H-TaS_2_ as 82 GPa as *E*^3D^ = *v*_p_^2^*ρ*(1 − *ν*^2^) where *ρ* = 6.86 g cm^−3^ is the density of 2H-TaS_2_ and *v*_p_ is the velocity of the quasi-longitudinal wave that has the highest propagation velocity. Thickness independence of Young's modulus from bulk down to a few layers can be explained by large shear strain energy of TaS_2_ layers.^2^ Another parameter we can extract from the eqn (1) by fitting the indentation data is the pretension *σ*^2D^_0_ of the suspended membrane. The values range from 1.2 N m^−1^ to 10.2 N m^−1^ in our case and the pretension is in an increasing trend with the crystal thickness (Fig. S9†). This shows a strong interaction between the membrane and the hole walls.

We also determined the breaking stress, *σ*^2D^_m_, and the breaking strength, *σ*_m_, using the equation 
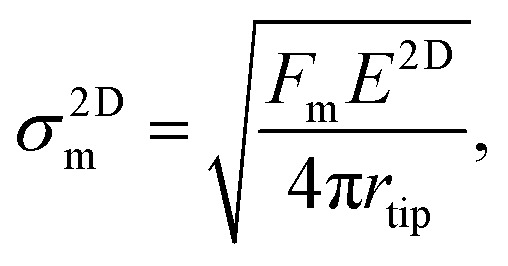
 derived based on the continuum model of a circular membrane in the elastic regime under a spherical indenter with radius *r*_tip_ and breaking force of *F*_m_.^1^ Loading and unloading of the membrane virtually repeating the same *F*–*δ* curve shows that the membranes are in the elastic regime (Fig. 2a). Here, the model assumes that 
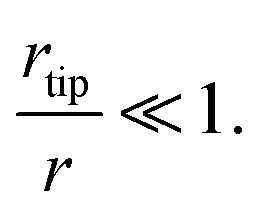
 However, measuring the *r*_tip_ is imperative due to our choice of stiff cantilever. We carefully characterized the AFM tips using SEM to ensure that we use the correct *r*_tip_ value for calculating the breaking strength. First, we measured the pristine tip radius, that turns out to be within the specifications of the manufacturer for all the tips we used. Then, we measured the tip radius after a scan over the crystal and after an indentation without breaking. This *r*_tip_ value is used for the breaking strength calculation. Side-by-side comparison of the SEM micrographs of the tips (Fig. 2b) show that the tips are slightly worn after each experiment to a certain extent. Some tips become significantly blunt after breaking the crystal, due to sudden strike of the tip to the base of the hole (Fig. S10†). Thus, we used each tip for a single breaking (and indentation) measurement. Fig. S11† shows a series of AFM tips used in scanning, indentation, and breaking experiments. We found that the tip radius is typically within *r*_tip_ = 22 ± 2 nm after each experiment. *σ*^2D^_m_ increases with the increasing crystal thickness with an average thickness independent breaking strength of 5.07 ± 0.10 GPa as shown in Fig. 2c. The breaking strength of 2H-TaS_2_ crystals are 6% of their Young's modulus. This is very close to the breaking strength/Young's modulus ratio that is reported for the few layer MoS_2_.^3^ The breaking strength is comparable to the monolayer MoS_2_ and WSe_2_ making 2H-TaS_2_ a considerable choice to form Schottky junctions for flexible electronics.^19^

**Fig. 2:**
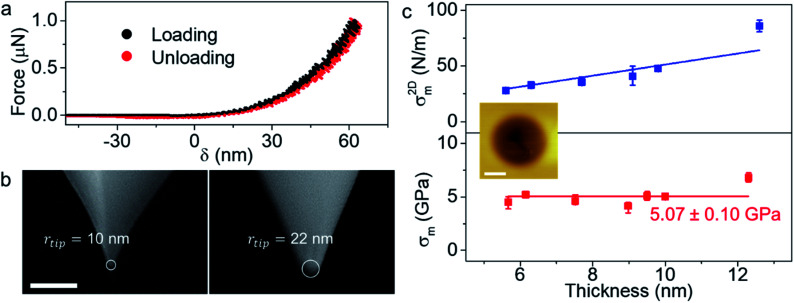
(a) Loading–unloading curve of the same crystal shows the elastic behavior of the membrane. ZDP is not corrected. (b) SEM images of the AFM tip before and after the indentation show that the tip radius almost doubles due to the wear. Scale bar is 100 nm. (c) Upper panel shows the 2D breaking strength of 2H-TaS_2_ membranes at different thicknesses with the AFM height map of a fractured crystal in the inset. Scale bar is 500 nm. Lower panel shows the ultimate strength of the material with an average of 5.07 ± 0.10 GPa.

We performed *ab initio* calculations at 0 K to gain a deeper understanding of our experimental results as well as to provide a validation to our measurements (details of the computational methods are provided in the ESI†^23–31^). We also performed experiments on three other thin crystals; 3R-NbS_2_, 1T-NbTe_2_ and 1T-TaTe_2_ to observe the general trend in metallic TMDCs. Fig. 3a and b show the crystal structures of the monolayers of various metallic TMDCs. All the crystal structures and crystal parameters are given in Fig. S12.† The breaking strength values are calculated *via* the strain *vs.* stress curves (Fig. 3c) which are obtained by stretching the monolayer crystals in biaxial direction. The deviation from the experimental data is possibly obtained due to the stacking fault, crystal orientation, and defect density which are not included in the calculations.^10^ The strain–stress curves are obtained by applying the strain up to 25%.

**Fig. 3:**
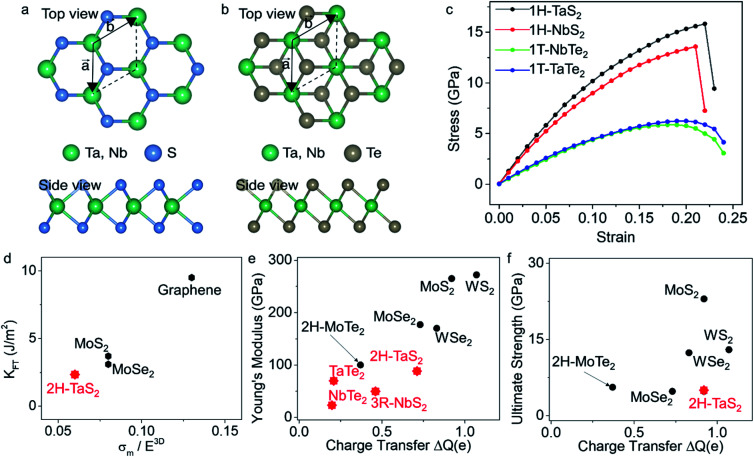
(a) and (b) show the top and side views of the 1H (sulfides) and 1T (tellurides) monolayers of the materials studied in this work. (c) Biaxial stress–strain curve calculated using DFT till the instability of the lattice. (d) Fracture toughness (*K*_FT_) *vs.* breaking strength/Young's modulus (*σ*_m_/*E*^3D^) ratio for various 2D layered materials. Graphene shows the highest fracture toughness with remarkably high *σ*_m_/*E*^3D^ ratio while the 2H-TaS_2_ we measured in this study exhibit a lower value in trend with other materials.^1,11,33^ (e) Experimentally determined Young's modulus *vs.* charge transfer is plotted in the figure for the metallic TMDCs. Charge transfer values are obtained *via ab initio* calculations for monolayer crystals. As the charge transfer from transition metal to chalcogen becomes larger, Young's modulus increases. (f) A similar trend is observed in the ultimate strength of the materials.^3,9–12,34^ Data point marked by red star represents the results from this work.

To compare the brittleness of the metallic TMDCs to other 2D layered materials, we calculated the surface energy, *ε*_BP_, in the basal plane. The edge energy of a monolayer is defined as 
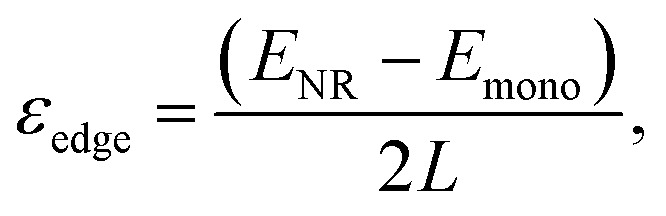
 where *E*_NR_ and *E*_mono_ are the total energy of the nanoribbon and the pristine monolayer (with the same number of atoms), respectively and, *L* is the ribbon length. In these calculations, the zigzag- and armchair-edged nanoribbons were constructed with 24 and 36 atoms, respectively. The surface energy was determined by the relation, *ε*_BP_ = *ε*_edge_*t*, where *t* is the thickness corresponding to the interplanar spacing. The model and the obtained results agree with the literature.^11,32^ Based on the lowest surface energy of the basal plane, *ε*_BP_, we can estimate the fracture toughness, *K*_FT_, of the materials. Here, we define the fracture toughness as the critical strain energy release rate of the fracture, as common in the literature.^7^ For brittle materials, the fracture toughness is roughly twice the surface energy of the basal plane.^11^ We obtained 1.14, 1.11, 0.39, and 0.35 J m^−2^ for the lowest surface energy of the basal plane of 2H-TaS_2_, 3R-NbS_2_, TaTe_2_, NbTe_2_ respectively. Fig. 3d shows *K*_FT_*vs. σ*_m_/*E*^3D^ for various 2D layered materials. An ideal material for high strength applications should have large ultimate strain and fracture toughness. Graphene shows exceptional mechanical properties whereas MoS_2_ and MoSe_2_ has higher fracture toughness than all four materials we studied in this work with lower *σ*_m_/*E*^3D^ ratio. These insights are relevant for engineering applications of these materials.

We investigated the bulk charge transfer in TMDCs studied here *via* DFT and compared the results to Mo and W based TMDCs. Mechanical properties of TMDCs are strongly influenced by the bonding charge distribution.^34^ DFT studies by Li *et al.*^34^ show that for the TMDCs, the Young's modulus and the ultimate strength of sulfides are the largest and selenides are larger than the tellurides with the same transition metal. The charge density between the chalcogen and the transition metal atoms decreases from sulfides to tellurides. This results in weakening of the covalent bonding in the basal plane. Our measurements on 2H- and 1T-crystals confirm the weakening of the covalent bonding in the basal plane. The charge transfer values (Δ*Q*) are correlated with the Young's moduli of these materials except 3R-crystal due to different stacking orders. Fig. 3e and f show the measured Young's moduli and the ultimate strength of various 2D layered materials *versus* the charge transfer values from DFT, respectively.

All the measured and calculated parameters for the metallic TMDCs studied here are listed in Table 1. The deviations from the experimental data, particularly for NbTe_2_, can be explained by the rapid oxidation of the NbTe_2_ surface leading to a decrease in the measured Young's modulus and breaking strength value.^35^ To test this hypothesis, we measured the oxidation of TaS_2_, TaTe_2_, NbTe_2_ and NbS_2_ surfaces using XPS. Freshly cleaved crystals of all materials show no signs oxidation while after one-hour in the ambient all materials except TaS_2_ show new peaks in both the metal and the oxygen XPS surveys that can be attributed to formation of metal-oxides (Fig. S13†). This measurement shows that metallic TMDCs oxidize faster under the ambient and extra care should be taken to prevent oxidation of samples during the indentation studies. Details of the XPS surveys are given in the ESI.† Rate of oxidation possibly plays a role in the measured values of the Young's modulus and the breaking strength. Rapidly oxidizing crystals show more significant deviations from the measured values.

**Table 1:** Measured and calculated parameters for the metallic TMDCs at room temperature. DFT results are given in italic

		2H-TaS_2_	1T-TaTe_2_	3R-NbS_2_	1T-NbTe_2_
Young's modulus (GPa) (*E*^3D^)	Measured	85.9 ± 10.6	70 ± 14	49.4 ± 3.0	23.6 ± 1.6
	DFT	*77.7*	*70.9*	*56.2*	*67.6*
Breaking strength (GPa) (*σ*_m_)	Measured	5.01 ± 0.10	7.18 ± 0.40	5.0 ± 1.5	2.9 ± 0.3
	DFT	*15.8*	*6.2*	*13.6*	*5.9*
Breaking strength/Young's modulus	Measured	0.06 ± 0.01	0.10 ± 0.03	0.10 ± 0.04	0.12 ± 0.02
Poisson's ratio (*ν*)	DFT	*0.27*	*0.10*	*0.27*	*0.11*
Number of samples measured		26	4	2	7

In conclusion, we reported the elastic and breaking properties of 2H-TaS_2_ and provided an insight into other layered Ta and Nb based metallic TMDCs. Our findings show that these materials can sustain large strains similar to semiconducting 2D materials and can be used in conjunction with other TMDCs as the electrical contact materials for flexible electronics and optoelectronics. The metallic TMDCs can endure similar maximum strain as the semiconducting TMDCs. All the materials we studied in this work are possible candidates as contact materials to the semiconducting TMDCs. Moreover, their strain dependent properties can be exploited in various applications *via* strain engineering.

## Availability of data

The data that support the findings of this study are available from the corresponding author upon reasonable request.

## Author contributions

TSK proposed and conducted the experiments. AS performed the indentation studies, prepared the samples and the substrates. NM prepared the samples, performed measurements on the crystals and aided the data analysis. IE performed the data analysis with the help of AS. HRR performed the XPS measurements. MMC and ED performed the *ab initio* studies. All authors contributed to the writing of the paper and discussed the results.

## Conflicts of interest

There are no conflicts to declare.

## Supplementary Material

NA-003-D1NA00225B-s001
