# Supplementary material for: High elasticity and strength of ultra-thin metallic transition metal dichalcogenides
Source: Nanoscale Adv. 2021 May 24;3(13):3894–9. doi: 10.1039/d1na00225b (PMC9419869; doi:10.1039/d1na00225b)
Supplement: NA-003-D1NA00225B-s001 [file NA-003-D1NA00225B-s001.pdf]

Electronic Supplementary Material (ESI) for Nanoscale Advances.
This journal is © The Royal Society of Chemistry 2021

Supporting Information: High elasticity and strength of ultra-thin metallic transition metal dichalcogenides

Ali Sheraz[1], Naveed Mehmood[2], Mert Miraç Çiçek[2,3], İbrahim Ergün[1], Hamid Reza Rasouli[2], Engin Durgun[2], T. Serkan Kasırga[1,2]

[1] Department of Physics, Bilkent University, Ankara, Turkey 06800
[2] Institute of Materials Science and Nanotechnology – UNAM, Bilkent University, Ankara, Turkey 06800
[3] Department of Engineering Physics, Faculty of Engineering, Ankara University, Ankara 06100, Turkey

Experimental Methods

We obtained bulk crystals from HQ Graphene and exfoliated using Nitto blue tape over polydimethylsiloxane (PDMS) stamps for the transfer. Then, using a deterministic dry transfer method, thin crystals are transferred over the holes drilled on SiO_2 substrate following Castellanos-Gomez et al.[1]. 3R-NbS_2 crystals are synthesized on sapphire substrate via salt-assisted chemical vapor deposition in a split-tube furnace. The growth recipe is provided in the following section. MFP-3D Asylum Research AFM is used for the indentation experiments. We used Tap300Al-G and Tap300DLC diamond-like coated tips from the Budget Sensors with force constant of 40 N/m. As mentioned in the main text, a dummy sample is scanned for about an hour before we conduct the indentation experiments on the real sample to limit the exposure of our samples to the ambient. Further scans are performed over the actual sample till the signs of drift disappears. During the indentation, Z_{Piezo} displacement speed was controlled at 100 nm/s to get hysteresis free loading and unloading curves. We have chosen to wrinkle free flakes with large surface area, uniformly suspended over the holes to obviate inaccuracy, hysteresis, and slippage of flakes.

Deflection (δ) of the suspended flake can be found from the deflection of AFM cantilever (ΔZ_C) and the scanning piezotube displacement (ΔZ_{Piezo}) through[2]:

$$\delta = \Delta Z_{Piezo} - \Delta Z_C$$

Force applied to suspended 2D flakes can be calculated using Hook`s law $F = K_C \, \Delta Z_C$ here K_C is deflection of AFM cantilever (40 Nm^{-1}) that we calibrated using GetReal calibration in Asylum AFM, which first performs the thermal noise spectrum measurement followed by the Sader's method calibration. Finally, the thermal noise method is used one more time to obtain the inverse optical lever sensitivity. This method is slightly better than the hard strike in preserving the cantilever tip.

Computational Methods

First-principles calculations based on density functional theory (DFT)[3,4] as implemented in the Vienna Ab Initio Simulation Package (VASP)[5] were performed. The Kohn-Sham equations were solved using the projector augmented-wave method[6] and the exchange-correction functional was described within the generalized gradient approximation (GGA)[7]. The kinetic-energy cutoff for plane wave basis set taken to be 550 eV and 15x15x1, 15x15x11, 9x15x11 Gamma-centered k-point mesh was used for the numerical integrations over the Brillouin zone for 2H-, 3R-, 1T-

crystals, respectively. The lattice constants and atomic positions were optimized until the total energy and force convergence were below 10^{-6} eV and 0.01 eV $Å^{-1}$, respectively. A vacuum space of 20 Å was taken along non-period direction to avoid the interactions between the neighboring images for monolayer crystals. The charge transfer analysis was performed by using Bader technique[8]. The mechanical response of monolayers in the elastic regime was determined by calculating the in-plane stiffness, $Y_{2D} = \frac{c_{11}^2 - c_{12}^2}{c_{11}^2}$, where the c_{ij}'s are the elastic constants including hydrostatic and shear terms[9]. The Young modulus of bulk crystals were calculated by using open-source code[10] based on Voigt-Reuss-Hill averaging scheme[11]. A denser k-point mesh was taken for calculations on mechanical properties.

CVD Growth of NbS_2 and Wet Transfer Method

NbS_2 flakes were grown using ambient pressure chemical vapor deposition method on a c-cut sapphire substrate. Before the growth, the sapphire substrates are cleaned using acetone, isopropanol, water and dried by blowing N_2 gas. Niobium pentoxide (Nb_2O_5) powder, crushed salt (NaCl) and Sulfur are used as the growth precursors. Nb_2O_5 and NaCl are milled together using mortar and pestle. The mixture is transferred in an alumina boat and the sapphire substrate is placed over the boat facing down at a few millimeters to the precursor powder. Sulphur precursor is placed in a different alumina boat. Both precursors are positioned in a quartz tube and the tube is slid into the tube furnace. **Figure S1** shows the schematic of the growth setup.

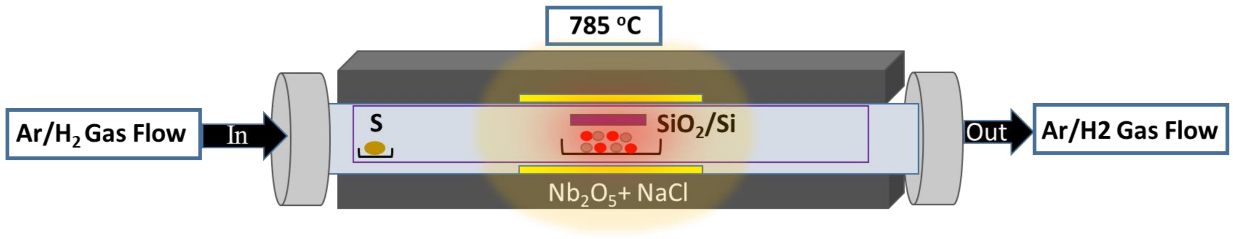

Figure S1 Schematic of CVD growth chamber for 3R-NbS_2 crystals showing different temperature zones and configuration of sample and precursors.

Once the chamber is sealed, we purged the chamber using Ar gas flow rate 499 sccm for 6 minutes to get rid of the air inside the chamber. After 6 minutes, we set Ar at 20 sccm flow rate and started the CVD chamber heater then waited for it to reach at 785 ^oC. After 785 ^oC is achieved, Ar and H_2 flow 100 sccm and 12 sccm respectively. After 10-15 minutes, we stopped the H_2 flow and set Ar again at 20 sccm during cool down. We got large area thin NbS_2 flakes which are exceedingly difficult to obtain via mechanical exfoliation. **Figure S2** shows some examples of the 3R-NbS_2 crystals on sapphire substrate.

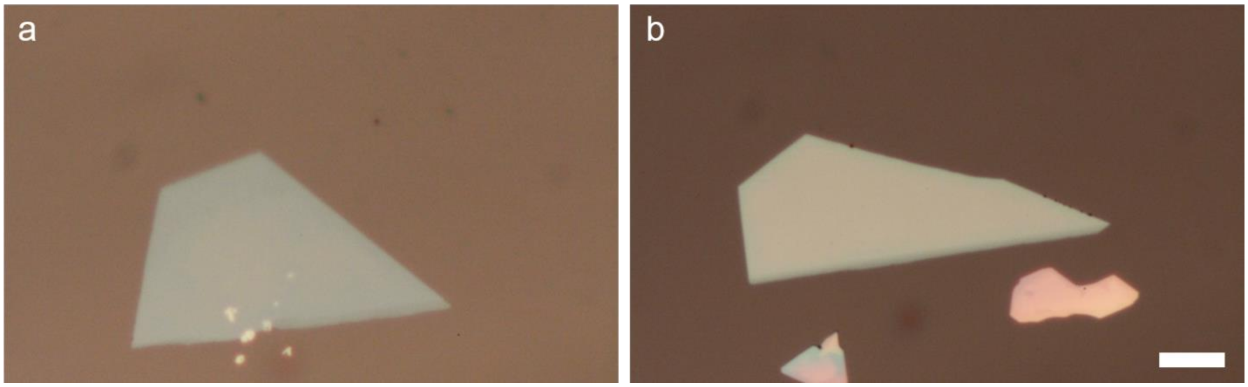

Figure S2 a and **b** show the optical microscope micrograph of 3R-NbS_2 thin crystals grown on c-cut sapphire via CVD. Scale bar is 10 µm.

XPS and EDX maps taken from the NbS_2 flakes show X-Ray Na intercalation within the crystals. **Figure S3** shows the XPS surveys for Nb, S and, Na after various etching cycles. We hypothesize that there might be Na intercalation between the layers as there are reports in the literature showing Na-NbS_2 crystals.

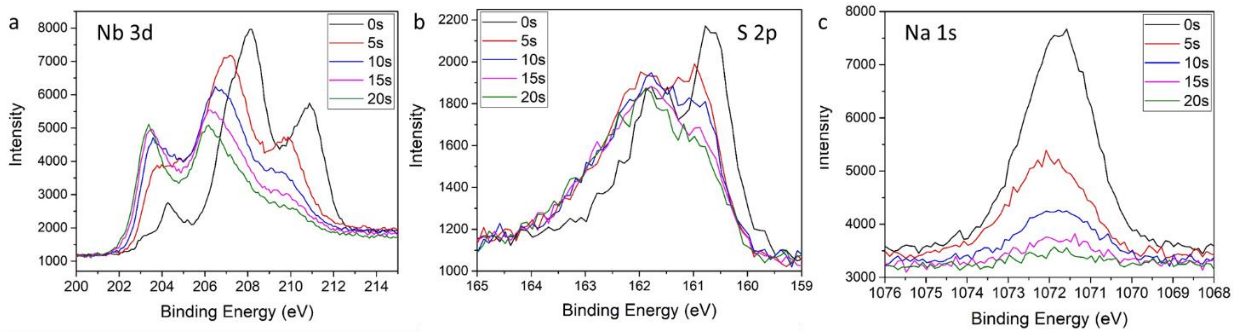

Figure S3 XPS spectra for **a.** Na 3d, **b.** S 2p and, **c.** Na 1s peaks after various etching cycles.

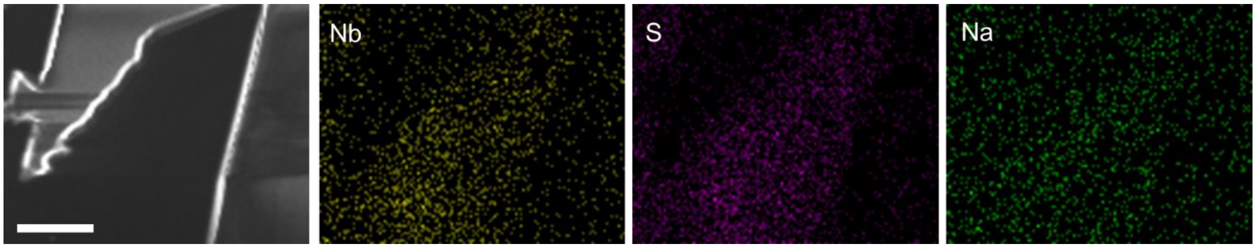

Figure S4 SEM image and corresponding EDX maps show the existence of Nb, S and Na in the crystal.

Wet Transfer of NbS_2 Crystals

Sapphire substrate with CVD grown NbS_2 is spin coated with Poly(methyl methacrylate) PMMA 495 (A4) at 1300rpm. The substrate is heated at 180 ^oC for 6 minutes. Then, the substrate is dipped into buffered oxide etch (BOE) for 20 minutes to release the PMMA film along with the CVD grown crystals from the substrate. After rinsing, the film is released onto water surface by

wedge transfer method and subsequently picked up by PDMS stamp. The PMMA film is dissolved in acetone for 20 minutes to transfer NbS_2 crystals successfully. Finally, the desired crystal was transferred onto the holes using deterministic dry transfer technique as mentioned in main text.

Raman Spectra of 2H-TaS_2, 3R-NbS_2, 1T-$TaTe_2$, 1T-$NbTe_2$

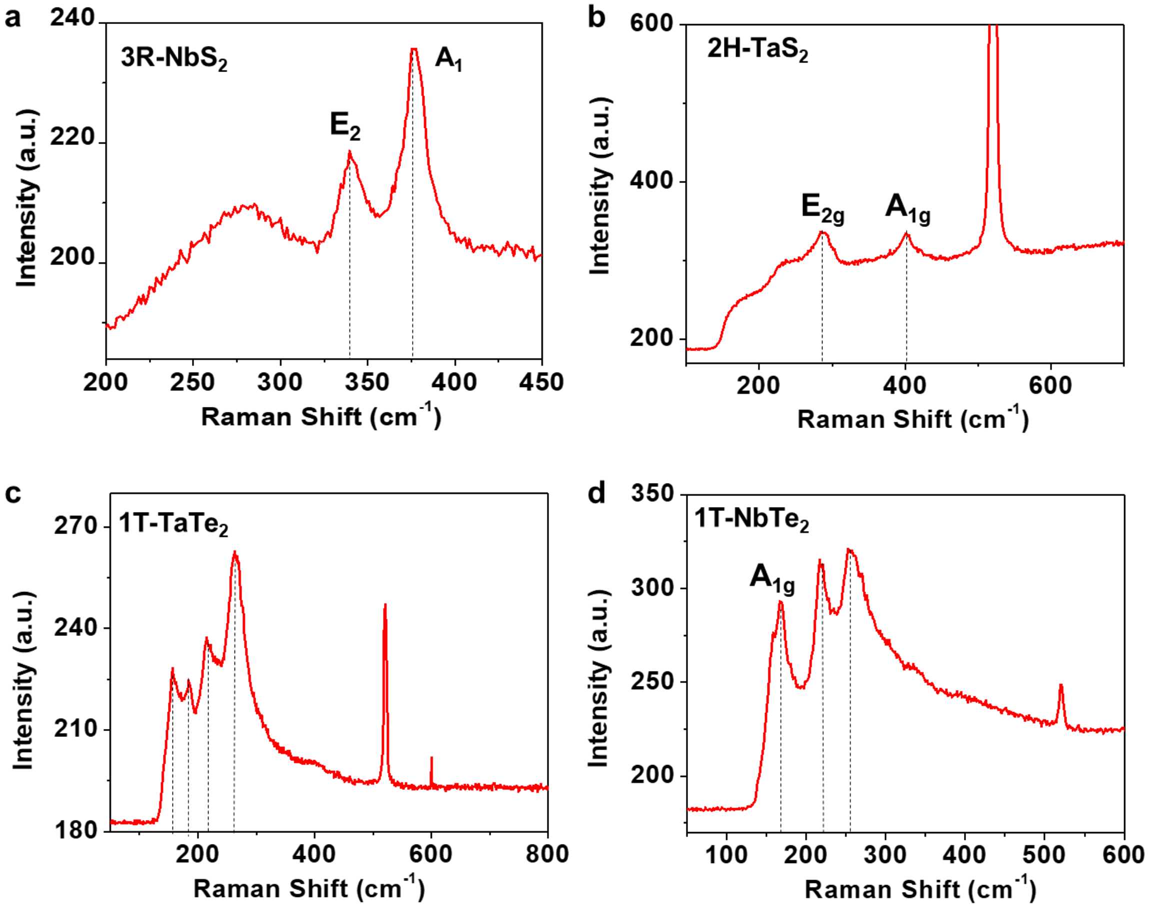

Figure S5 Raman spectra taken from the crystals studied in this work. **a.** Raman spectrum of 3R-NbS_2 **b.** 2H-TaS_2 **c.** $TaTe_2$ and **d.** $NbTe_2$. Identified modes are labelled on the corresponding peaks. Dashed vertical lines show the matching peaks with the literature.

We performed Raman spectroscopy to confirm the phase of the crystals studied in this work. **Figure S5** shows the exemplary Raman spectra we obtained for each crystal. Dashed lines indicated on the spectra shows the matching lines with the literature[12,13]. Although the Raman spectrum of NbS_2 matches with the spectrum reported for 3R-NbS_2 in the literature, as we discuss in the following sections, X-ray photoelectron spectroscopy (XPS) and energy dispersive

spectroscopy (XPS) shows that the NbS_2 crystals we have might have Na atoms interstitially between the layers.

Exemplary Optical Microscope Micrographs of Suspended Crystals

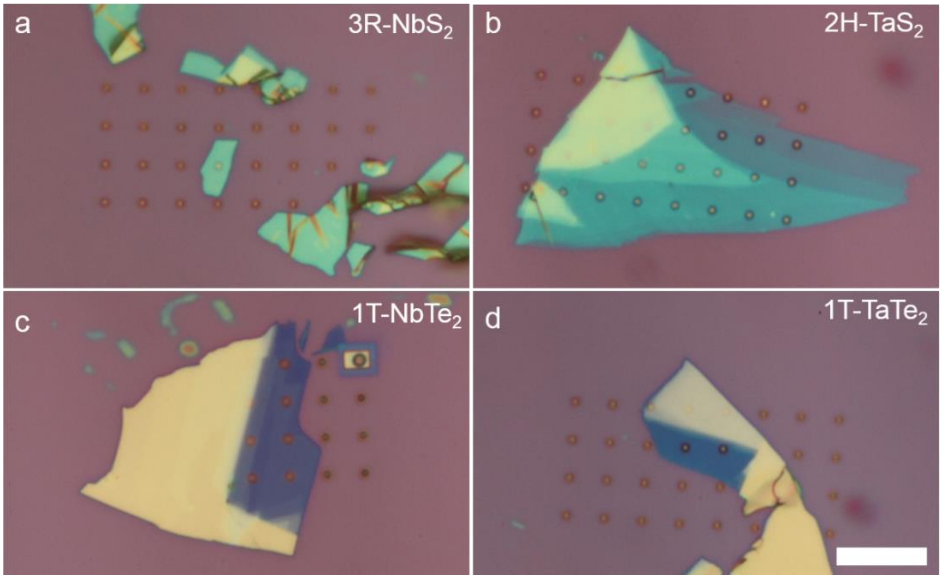

Figure S6 Optical microscope micrograph of **a.** 3R-NbS_2 **b.** 2H-TaS_2 **c.** 1T-$NbTe_2$ and **d.** 1T-$TaTe_2$. Scale bar is 10 µm.

Calibration of AFM Probes using GetReal Calibration Method

We used built-in GetReal method for automated calibration of AFM probes that uses Sader's method[14] and thermal noise method for the calibration of cantilever's spring constant and sensitivity (InvOLS) respectively.

Force-Indentation Loading Curve

A full cycle of the $F - \delta$ loading curve is given in **Figure S7**. Important points in the curve are marked on the graph. First the tip starts above the membrane. When the tip is sufficiently close, the tip snaps onto the membrane and indentation continues till the mechanical failure of the film. Then, the tip rapidly descends to the bottom of the hole and starts applying force there again.

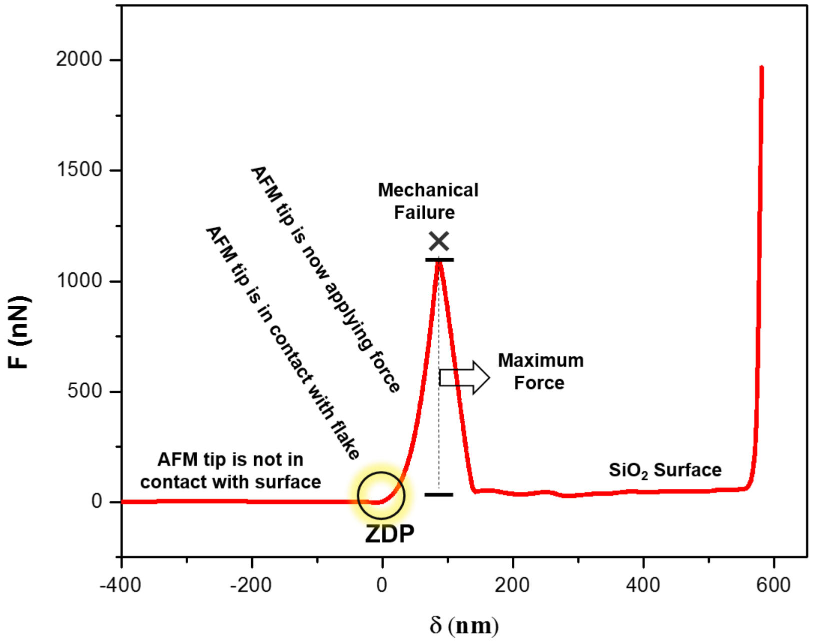

Figure S7. Figure shows the complete $F - \delta$ graph for a typical crystal reported in this work. Upon lowering the tip in the close vicinity of the suspended membrane, the membrane snaps to the tip around the zero-displacement point (ZPD). Then, further lowering the tip stretches the membrane till the brittle mechanical failure. Then, the tip reaches down to the bottom of the hole till it touches the SiO_2 surface.

Individual Data Points from 2H-TaS_2 Samples

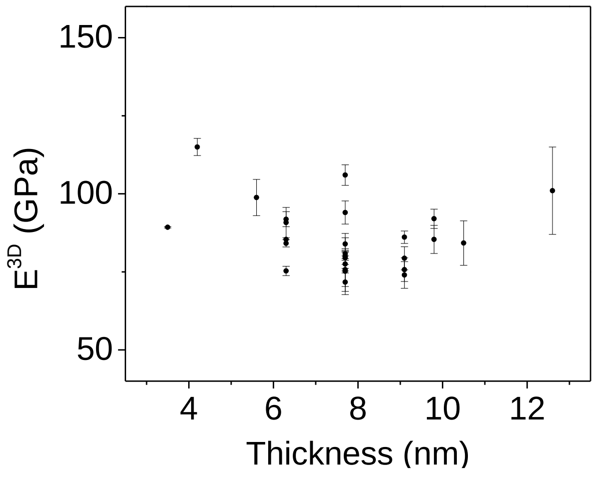

Figure S8 Set of individual data points over different samples of 2H-TaS_2. **Figure 1d** reported in the main text is the averaged values of these data points.

Pretension and Prestress of 2H-TaS_2

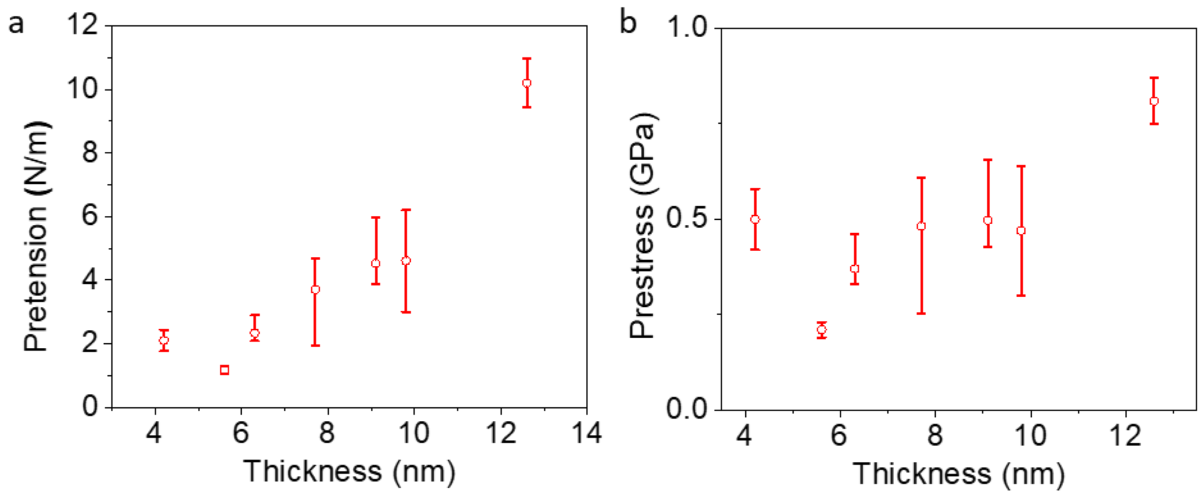

Figure S9 a. Pre-tension and **b.** pre-stress values for 2H-TaS_2 crystals of various thickness are shown.

SEM Micrographs of AFM tips After a Single Measurement

We characterized the changes on a single AFM tip using SEM imaging. We first acquired SEM image of a pristine tip. Then, scanned the sample and took another SEM image. We installed the same tip to the AFM, this time we scanned the sample and performed an indentation and took an SEM image. Finally, we used the same tip for a scan, indentation and breaking and acquired an SEM image. **Figure S10** shows the series of SEM images after each step and gradual degradation of the AFM tip.

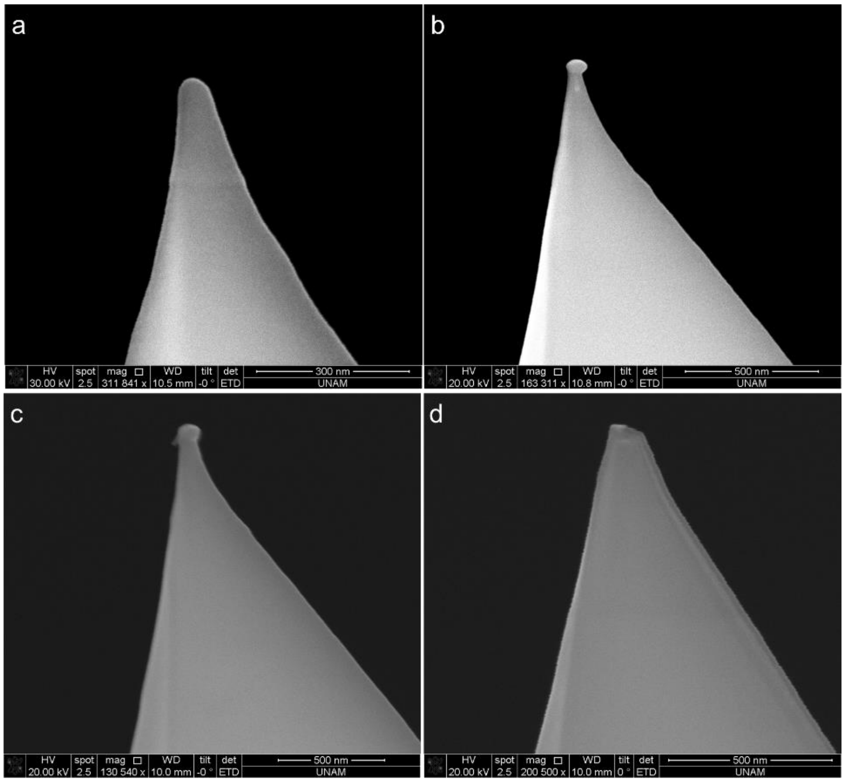

Figure S10 a. SEM micrograph of a pristine tip. The tip radius is measured as 12 nm. **b.** SEM micrograph of the same tip in **a** after a single scan typically performed to map the crystal. Tip radius is 23 nm. **c.** Same tip in **b** after a second scan followed by an indentation. Tip radius is 28 nm. **d.** Same tip in **c** after a third scan, indentation and a breaking. Tip is now blunt.

Before the measurements we picked up 10 random tips from the box and measured the diameter of the AFM tips. We found out that the tip radii are within the specifications of the manufacturer (10.0 ± 1.0 nm). We used each tip for a single indentation and breaking measurement and after every other measurement we measured the tip radius using SEM imaging (**Figure S11**). For the measurements we have the measured tip value, we used the measured value, otherwise we used the averaged tip radius 22.0 ± 2.0 nm.

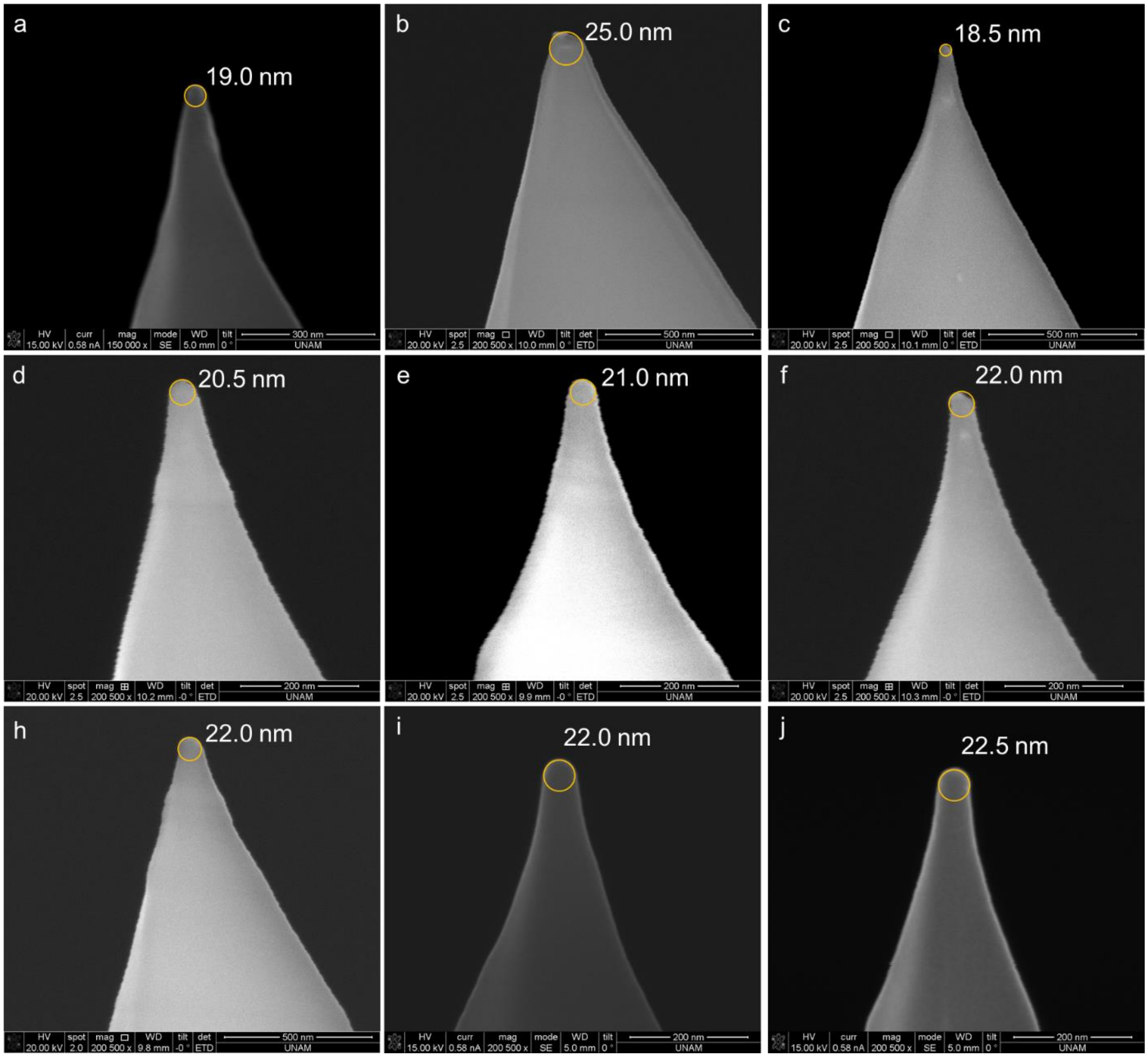

Figure S11 SEM micrographs of DLC AFM tips after a single indentation and breaking measurement. The yellow circles show the tip apex, and the values are the tip radii.

Crystal Structures of 2H-TaS_2, 1T-$TaTe_2$, 3R-NbS_2, 1T-$NbTe_2$

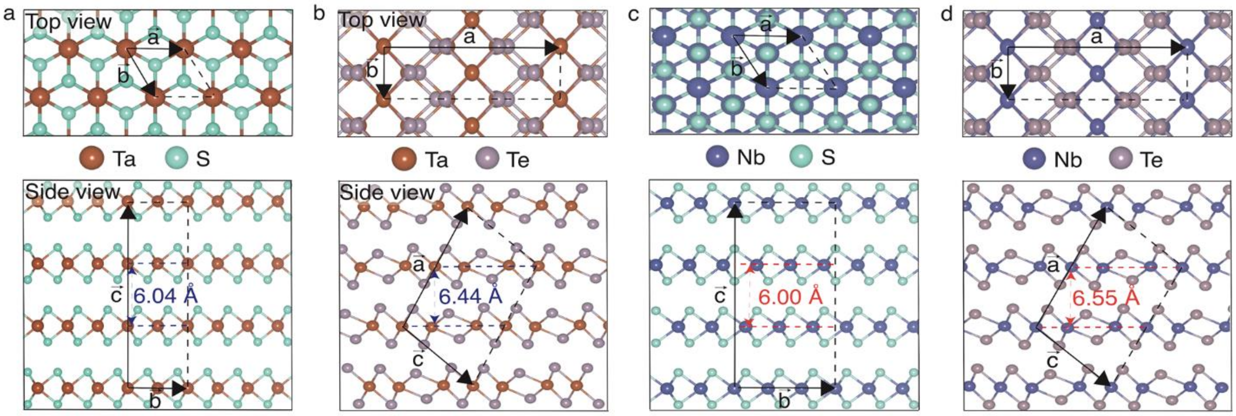

Figure S12 a. 2H-TaS_2 **b.** 1T-$TaTe_2$ **c.** 3R-NbS_2 **d.** 1T-$NbTe_2$

XPS Survey on bulk metallic TMDCs

We performed XPS surveys on exfoliated TDMCs right after exfoliation and after keeping the samples under ambient for an hour. Except TaS_2 other metallic TMDCs showed significant signs of oxidation. **Figure S13** shows XPS surveys of the chalcogen and metal constituents of the metallic TMDCs.

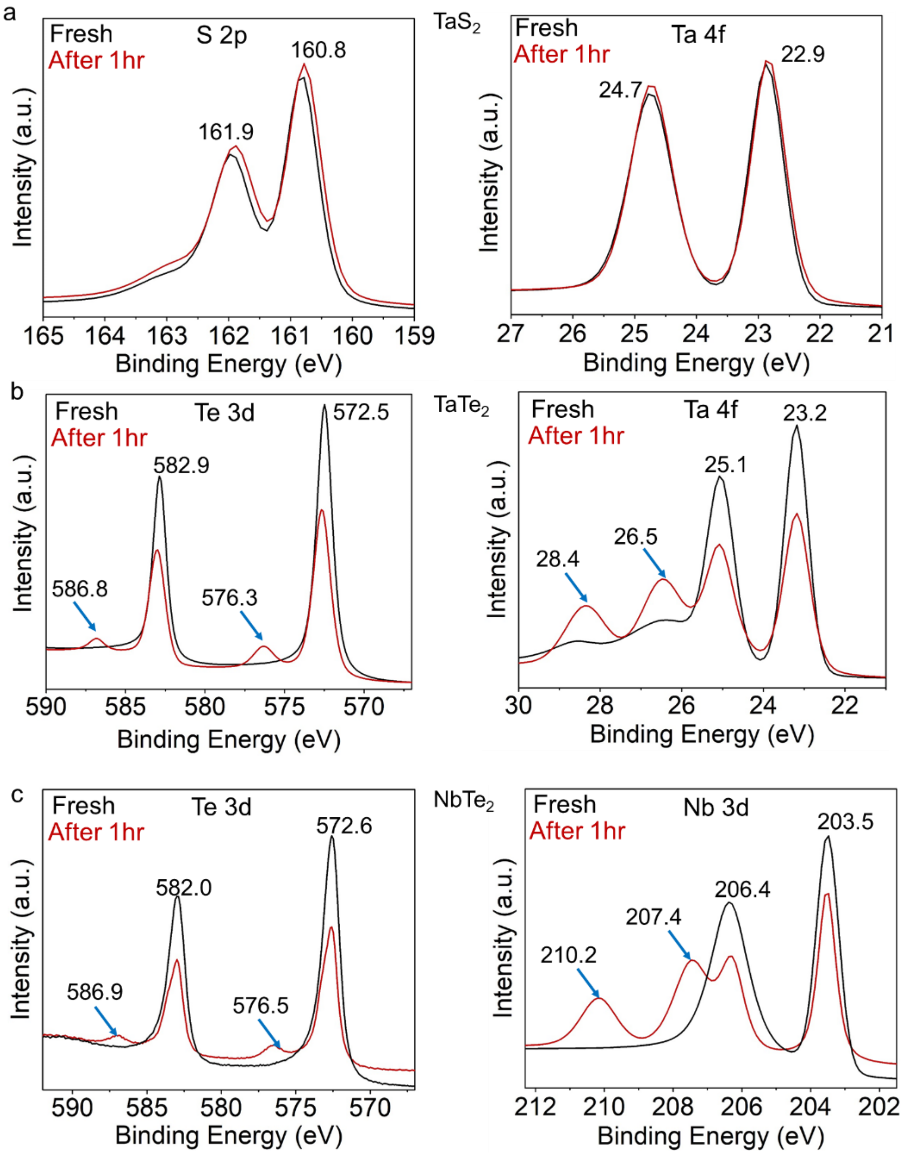

Figure S13 a. S 2p and Ta 4f surveys of TaS_2 taken on freshly exfoliated samples and after exposing to ambient for 1 hour. **b.** Te 3d and Ta 4f surveys of $TaTe_2$ taken on freshly exfoliated samples and after exposing to ambient for 1 hour. **c.** Te 3d and Nb 3d surveys of $NbTe_2$ taken on freshly exfoliated samples and after exposing to ambient for 1 hour. $TaTe_2$ and $NbTe_2$ show clear signs of oxidation after 1 hour in ambient conditions.

References

(1) Castellanos-Gomez, A.; Buscema, M.; Molenaar, R.; Singh, V.; Janssen, L.; van der Zant, H. S. J.; Steele, G. A. Deterministic Transfer of Two-Dimensional Materials by All-Dry Viscoelastic Stamping. *2D Mater.* **2014**, *1* (1), 011002. https://doi.org/10.1088/2053-1583/1/1/011002.

(2) Frank, I. W.; Tanenbaum, D. M.; van der Zande, A. M.; McEuen, P. L. Mechanical Properties of Suspended Graphene Sheets. *J. Vac. Sci. Technol. B Microelectron. Nanom. Struct.* **2007**, *25* (6), 2558. https://doi.org/10.1116/1.2789446.

(3) Hohenberg, P.; Kohn, W. Inhomogeneous Electron Gas. *Phys. Rev.* **1964**, *136* (3B), B864–B871. https://doi.org/10.1103/PhysRev.136.B864.

(4) Kohn, W.; Sham, L. J. Self-Consistent Equations Including Exchange and Correlation Effects. *Phys. Rev.* **1965**, *140* (4A), A1133–A1138. https://doi.org/10.1103/PhysRev.140.A1133.

(5) Kresse, G.; Furthmüller, J. Efficiency of Ab-Initio Total Energy Calculations for Metals and Semiconductors Using a Plane-Wave Basis Set. *Comput. Mater. Sci.* **1996**, *6* (1), 15–50. https://doi.org/10.1016/0927-0256(96)00008-0.

(6) Blöchl, P. E. Projector Augmented-Wave Method. *Phys. Rev. B* **1994**, *50* (24), 17953–17979. https://doi.org/10.1103/PhysRevB.50.17953.

(7) Perdew, J. P.; Burke, K.; Ernzerhof, M. Generalized Gradient Approximation Made Simple. *Phys. Rev. Lett.* **1996**, *77* (18), 3865–3868. https://doi.org/10.1103/PhysRevLett.77.3865.

(8) Henkelman, G.; Arnaldsson, A.; Jónsson, H. A Fast and Robust Algorithm for Bader Decomposition of Charge Density. *Comput. Mater. Sci.* **2006**, *36* (3), 354–360. https://doi.org/10.1016/j.commatsci.2005.04.010.

(9) Kecik, D.; Onen, A.; Konuk, M.; Gürbüz, E.; Ersan, F.; Cahangirov, S.; Aktürk, E.; Durgun, E.; Ciraci, S. Fundamentals, Progress, and Future Directions of Nitride-Based Semiconductors and Their Composites in Two-Dimensional Limit: A First-Principles Perspective to Recent Synthesis. *Appl. Phys. Rev.* **2018**, *5* (1), 011105. https://doi.org/10.1063/1.4990377.

(10) Singh, S.; Valencia-Jaime, I.; Pavlic, O.; Romero, A. H. Elastic, Mechanical, and Thermodynamic Properties of Bi-Sb Binaries: Effect of Spin-Orbit Coupling. *Phys. Rev. B* **2018**, *97* (5), 054108. https://doi.org/10.1103/PhysRevB.97.054108.

(11) Hill, R. The Elastic Behaviour of a Crystalline Aggregate. *Proc. Phys. Soc. Sect. A* **1952**, *65* (5), 349–354. https://doi.org/10.1088/0370-1298/65/5/307.

(12) Nakashima, S.; Tokuda, Y.; Mitsuishi, A.; Aoki, R.; Hamaue, Y. Raman Scattering from 2H-NbS2 and Intercalated NbS2. *Solid State Commun.* **1982**, *42* (8), 601–604. https://doi.org/10.1016/0038-1098(82)90617-2.

(13) Hangyo, M.; Nakashima, S.-I.; Mitsuishi, A. Raman Spectroscopic Studies of MX2-Type Layered Compounds. *Ferroelectrics* **1983**, *52* (1), 151–159. https://doi.org/10.1080/00150198308208248.

(14) Sader, J. E.; Sanelli, J. A.; Adamson, B. D.; Monty, J. P.; Wei, X.; Crawford, S. A.; Friend, J. R.; Marusic, I.; Mulvaney, P.; Bieske, E. J. Spring Constant Calibration of Atomic Force Microscope Cantilevers of Arbitrary Shape. *Rev. Sci. Instrum.* **2012**, *83* (10). https://doi.org/10.1063/1.4757398.
